# Supplementary figures and images for: Paired-end mappability of transposable elements in the human genome
Source: Mob DNA. 2019 Jul 10;10:29. doi: 10.1186/s13100-019-0172-5 (PMC6617613; doi:10.1186/s13100-019-0172-5)

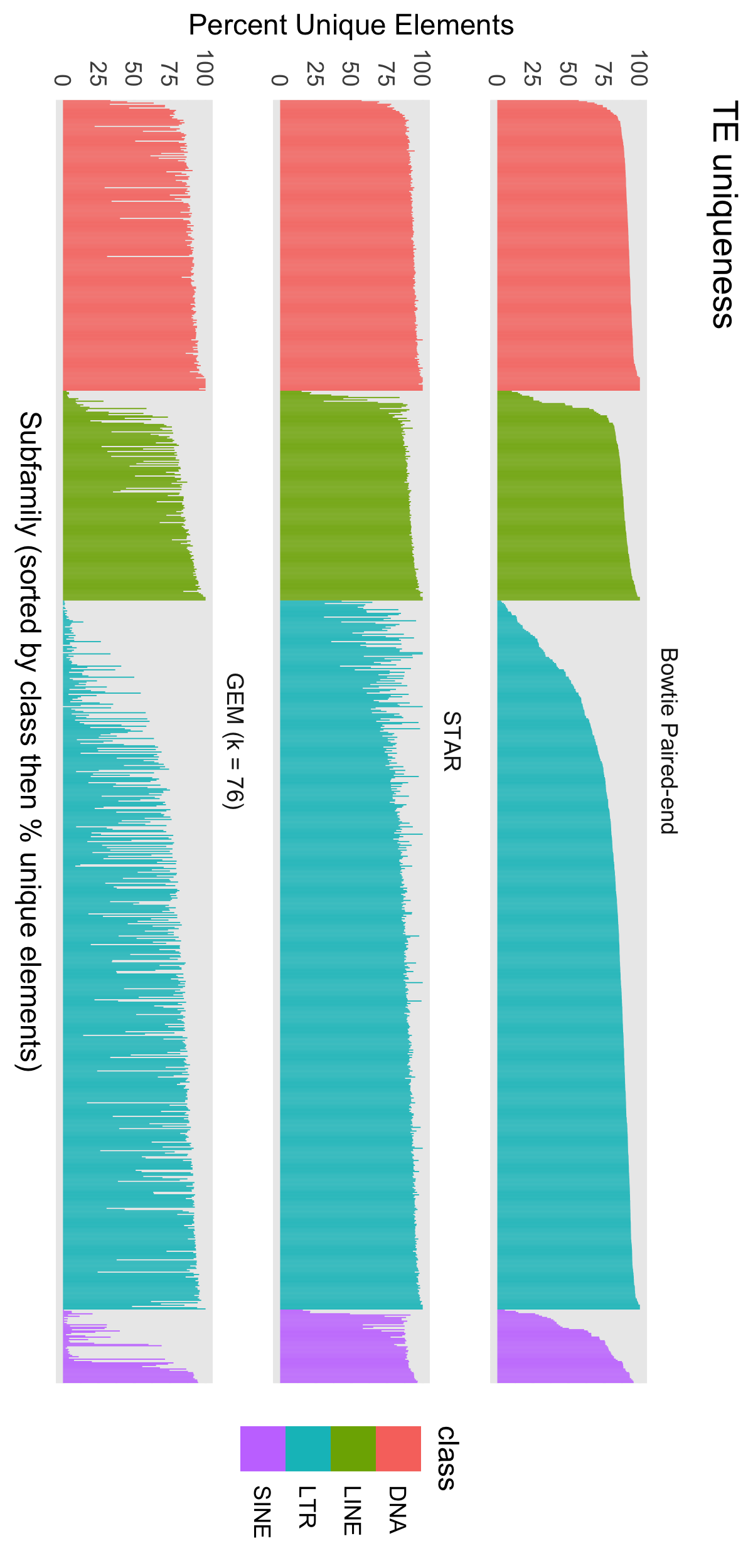

Supplement: Supplementary file 1 — Figure S1. Colored by class distinction, each bar represents the percent unique elements in each subfamily. An element is only considered unique if every position in its sequence has a unique mappability score. Both the Bowtie and STAR scores were generated by 76bp paired-end simulated kmers. (PNG 269 kb) [file 13100_2019_172_MOESM1_ESM.png]

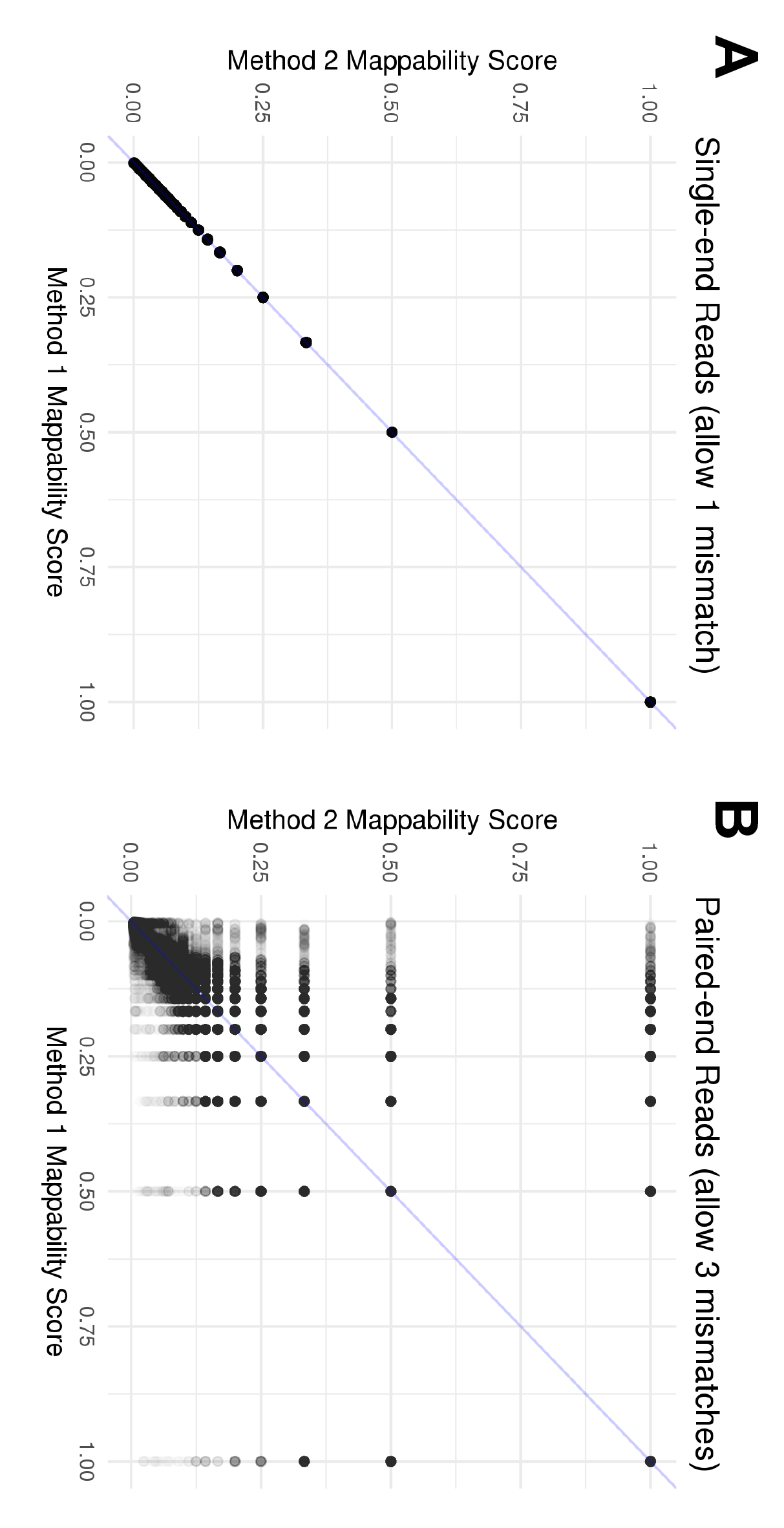

Supplement: Supplementary file 2 — Figure S2. Bowtie mappability calculation method comparison. Comparison of mappability scores when calculated with two different methods. Method 1: For a single base position, if a kmer mapped to that position and jellyfish (the kmer generator) reported that kmer to appear 5 times in the genome, the mappability of that position would be 1/5. Method 2: Identify a kmer which maps exactly to a base position and take the inverse of how many times that kmer appears at other positions in the bamfile. Although the figure does not reflect it very well, over 90% of the points are placed along the diagonal of the plot. In this study we use method 1, the more conservative measure, for all paired-end score calculations. (PNG 288 kb) [file 13100_2019_172_MOESM2_ESM.png]

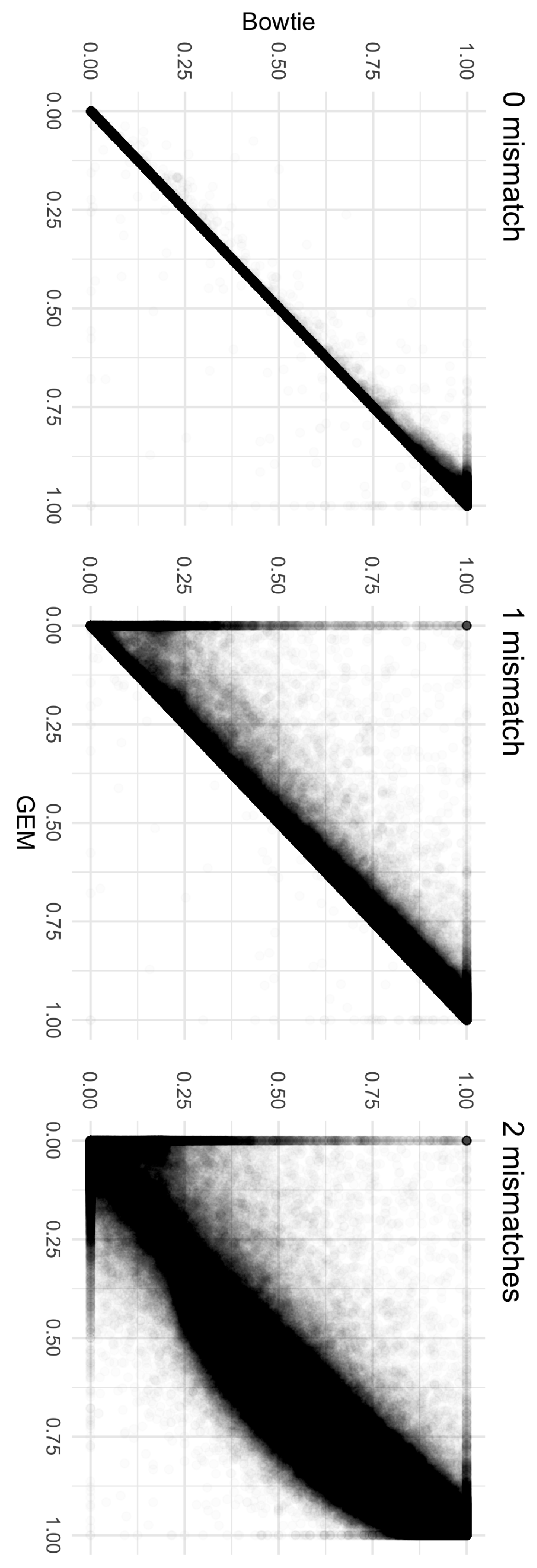

Supplement: Supplementary file 3 — Figure S3. Bowtie vs GEM Mappability scores for single-end mappability. Comparison of Bowtie 76bp mapping and GEM Mappability 76-mer mappability scores with 0, 1, and 2 mismatches. (PNG 849 kb) [file 13100_2019_172_MOESM3_ESM.png]

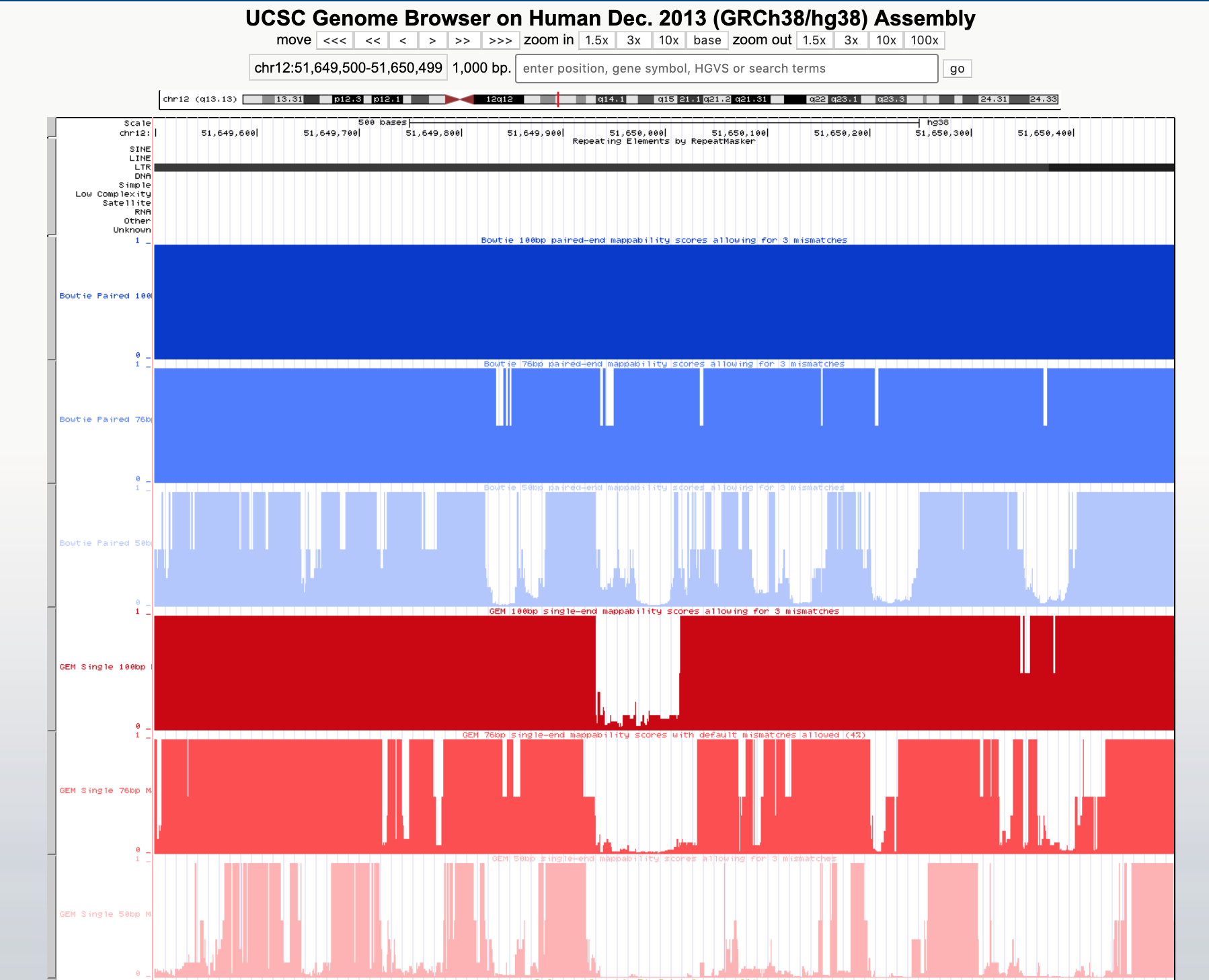

Supplement: Supplementary file 4 — Figure S4. UCSC Genome Browser tracks. Visualization of the 6 new hg38 UCSC Genome Browser tracks. The paired-end scores are in blue and the single-end scores are in red. (PNG 285 kb) [file 13100_2019_172_MOESM4_ESM.png]
